# Behavioral Economics, Motivating Psycho-Education Improvements: A Mobile Technology Initiative in South Africa

**DOI:** 10.3389/fpsyg.2019.01560

**Published:** 2019-07-10

**Authors:** Alexandra Mary Forsythe, Catherine Venter

**Affiliations:** ^1^Centre for Psychological Research, University of Wolverhampton, Wolverhampton, United Kingdom; ^2^School of Psychology, University of Liverpool, Liverpool, United Kingdom

**Keywords:** financial incentive, mobile app engagement, motivation orientation, psycho-education, mindfulness

## Abstract

Here we report on a health behavioral support project, using incentivized behavior on a mobile platform through M4JAM. This was a proof of concept study to support further developments, more specifically targeted at the management of tuberculosis and human immunodeficiency virus. The study reported here examines the impact of financial rewards and app toward improving mental health outcomes in South Africa. A total of 136 participants were recruited from a database and dichotomized into self-determined and heteronomous groups based on self-report scores. Overall the findings reported here highlight that personal financial incentives have a role in motivating behavior. The findings are discussed in light of the usefulness of an incentivized mobile platform in real-world practice to encourage mental health improvements in low- to middle-income countries.

## Mental Health in South Africa

The World Health Organization (WHO) maintains that the financial burden of neurological and mental health disorders is universal, with low- and middle-income countries the most challenged (World Health Organization, 2014). South Africa is no exception; it faces lack of community resource, human resources obstacles, limited funding, and a population where over 40% of people are living with human immunodeficiency virus and/or a diagnosable mental disorder. Cape Mental Health (2015) purports that one in six of the general population will experience a mental health problem, that there is an increased risk of depression, anxiety, and stress at middle age (35–59), and that more women than men will seek help. Bloomberg ranked South Africa as the second most stressed nation in the world, describing it as in a sick state of mental health, with an escalating rate of anxiety, depression, substance abuse, and suicide (Chiumia and van Wyk, 2014; The South African Federation for Mental Health, 2015). As a stopgap to the exigency of worldwide mental, neurological, and substance abuse disorders, WHO launched the Mental Health Gap Action Programme (mhGAP) in 2008 followed by the WHO’s Comprehensive Mental Health Action Plan 2013–2020.

Considering the mental health perplexity in South Africa and following WHO recommendations in regard to income generation and educational opportunities, the research project reported here investigates whether an incentivized psycho-educational initiative on a mobile device could have a positive effect with regard to the improvement and promotion of mental health, and if such an initiative could affect a difference between incentivized and de-incentivized groups.

There is compelling evidence for the efficacy of the integration of incentivized based programs (pay-for-performance) in healthcare on the modification of -damaging behavior, specific to alcohol consumption, diet, physical activity, sexual behavior and smoking (Lagarde et al., 2007; Dadich, 2010; Cahill and Perera, 2011; Betty, 2013; Stephens, 2014). In light that mobile phone penetration in South Africa is 133% and smartphone penetration is 47%, the ubiquity and exponential growth of mobile technology is not dissimilar to the mobile revolution evident in other low- and middle-income countries and supports the potential benefits of innovative initiatives for future improvements in mental health care (Davey and Davey, 2014).

### Mobile Technology: A Real-World Vehicle for Psychology Delivery

There has been a rapid increase in the use of interactive mobile technologies to communicate health behavior risks and accelerate behavior change (Cohn et al., 2011), in psycho-education (Cohn et al., 2011), stress management (Serino et al., 2014) and brief evidence-based positive psychology interventions (Howells et al., 2014).

Users are engaging actively with this technology, and the evidence from such studies has led to building a compelling case for the expansion of mobile technology in the European Union’s public health care strategy (Lalmas et al., 2015). Epstein and Bequette (2013) have challenged the efficacy of software technologies to be as effective as face-to-face therapy with the advantage that computerized psychotherapy is self-managed, more convenient, and non-judgmental. The authors also included the caveats of using cellular phones, namely the cost of the mobile device, the cost of Internet connectivity, the risk of loss or theft of device and threat to user’s confidentiality and privacy issues. However, with the rapid growth of mobile telephony technology and the exponential increase of smartphone users specifically in LAMICs, cell phones are considered as a logical extension for clinical practice and a useful tool for underserved persons who might not necessarily have access to psychotherapy (Svoboda and Richards, 2009; Morris et al., 2010; Aguilera and Muñoz, 2011). Dicianno et al.’s (2016) perspective article purports the unique functionality of mobile health technologies that support self-directed learning and novel applications. Mobile health (m-health) technology is a high-reach, low-cost solution for health care, not intended to replace but rather augment medical care. A review by Davey and Davey (2014) noted the potential of m-health strategies on mobile devices in LAMICs, as a practical solution for improving health outcomes in underserved locations. However, the attrition rate in e-studies remains as much of a challenge as in the field of behavioral economics generally. Recent work of Sonntag and Zizzo’s (2015) and earlier work by Frick et al. (2001) consistently found a higher attrition rate in e-studies compared with traditional settings.

To date, the application of large-scale incentivized psycho-education intervention by employing mobile technology requires further investigation. Here we seek to combine behavior economic principles and positive psychology, for the promotion of mental health in South Africa using advanced mobile software application. The current study replicates and extends existing smartphone-based research by Howells et al. (2014) who demonstrated the viability of delivering brief, evidence-based, positive psychology interventions on a mobile platform using a smartphone application, with the aim of boosting happiness and improving wellbeing. Their real-world approach was an attempt to showcase smartphone methodologies as a feasible tool and valid platform to deliver positive interventions.

### Financial Incentives to Support Behavior Modification

Application of the principles of behavioral economics in psychology is evident in the work of Abraham et al. (2011) and Haff et al. (2015). Abraham et al. investigated the factors that influence decision-making concluding that the low response rates that are indicative of incentive designs making the interpretation of findings subjective at best. Positive outcome-based incentive effects were however reported in the literature by Volpp et al. (2011) and Violino (2012). Violino reported a positive impact in their application in American community colleges, which led to the promotion of student success and improved institutional performances. Volpp et al. (2011) identified the use of BE in the life and health insurance sectors, finding that motivational tactics addressed health behavior challenges and reduced health care costs.

It is evident that BE has become a rapidly emerging discipline with broad ramifications in health practice and policies. Literature suggests that incentivized-based programs (IBPs) have the potential to influence human decision-making; however, it questions if IBPs undermine autonomous and intrinsic motivation.

The early literature of Lepper et al. (1973) reflects the traditional, broad-based assumption that extrinsic motivation is secondary to instinctive motivation and that outcomes motivated by intrinsic rather extrinsic forces are always more valued, concluding that there can be an “*undesirable consequence of the unnecessary use of extrinsic rewards*” (p. 135). Such sentiment was echoed in the work of Kohn (1993) and Pfeffer (1998). Further studies investigating the undermining effect (also referred to as motivation crowding-out effect) showed less volitional engagement for incentivized behavior (Deci and Ryan, 1985; Kirk, 1995; Deci et al., 1999). However, Camerer and Hogarth’s (1999) meta-analysis of performance-based incentives challenged such opinions and found that the effect of incentives was inconsistent and complicated.

Similarly, Fang and Gerhart (2012) found no conclusive evidence to support arguments of detrimental consequences of providing financial incentives to improve motivation and suggested that under certain conditions, incentives had a positive effect rather than a negative impact on levels of perceived autonomy, perceived competence and intrinsic interest.

In an attempt to bestow some finality Cerasoli et al. (2014), undertook nine meta-analyses, expanding 40 years of research involving (*k* = 183, *N* = 212, 468). They focused on the interrelationships of motivation and performance in organizations, education and physical domains and concluded that the impact of incentives was not consistent and proposed that intrinsic motivation and incentives influence performance together. They also concluded that there is a joint contribution of intrinsic and extrinsic motivation on performance; motivation is multifaceted; intrinsic motivation is deemed a superior performance determinant; and, that incentives can positively affect intrinsic motivation depending on the perceived value of the incentive. The authors recommended that policymakers consider compensation strategy designs in the future and incentivize less enjoyable tasks.

A broader investigation of IBP effectiveness for behavior change in the general population by Lynagh et al. (2013) identified that the type of incentive, incentive size, and the impact of timing of a stimulus are related to target population. Evidence was drawn from a careful non-systematic review of published literature (2009–2013) and suggested that negative incentives (penalties) are less efficacious in changing behavior than positive reinforcement. Data were drawn from American and British research with little evidence of long-term sustainability.

Roberts and Bailey’s (2013) ethnographic study, using qualitative methods, found that individuals with severe mental illness (SMI) are less likely to be motivated by financial incentives compared to the general population. These findings opposed “a one size fits all approach” and are consistent with the academic opinions of Sutherland et al. (2008); however, Roberts and Bailey’s study was limited by a small sample size (*N* = 8) and drawn from a single community center. A somewhat larger study examining a psycho-education program (Joice and Mercer, 2010) indicated efficacy in the short term for a mental health intervention without incentives for mild to moderate mental health problems. Findings suggest a potential to alleviate waiting list issues. However, the sample group was predominantly employed, white women, (31–60), and the attrition rate was high.

The most extensive study to date evaluated multiple sources of data from seven studies (Haff et al., 2015). Meta-analysis and random effects modeling suggest that the role of financial incentives in behavior modification is in line with what other authors had predicted; there was a definite incentive effect on health behavior, yet no consistent relationship between financial incentive and observable demographic characteristics.

## Methodology

Money for jam (M4JAM) is an accredited micro-jobbing mobile technology platform in South Africa, enabling registered members to earn money and vouchers on smartphone devices, (mobile wallets) by completing micro jobs. Typically, micro jobs involve market research surveys, mystery shopping, merchandising, brand activation, brand engagement, and point of interest validations. M4JAM was launched on the weChat platform in South Africa in 2014. M4JAM’s support for the current study was based on a preliminary proof of concept to test the effect of incentivized health behavior to support their interaction with the Gates Foundation in the screening and management of tuberculosis and human immunodeficiency virus. M4JAM had the technological expertise to develop the software required for the current study strategy and offered access to recruit potential participants from the registered M4JAM database. Further information about M4JAM can be found at http://www.m4jam.com.

As a proof of concept, the subject matter for the incentivized behavior for this study was mindfulness practice with positive psychology. There is strong empirical support for the application of positive psychology and mindfulness in self-regulation and wellness (Ryan and Deci, 2001; Brown and Ryan, 2003; Ryan, 2009). Such reflective awareness facilitates autonomy and growth tendencies apparent in intrinsic motivation and is incorporated in self-determination theory. Past research by Keune and Forintos (2010) on the usefulness of mindfulness meditation on subjective well-being found that mindfulness correlated with positive affect and vitality, and demonstrated that a relationship existed between mindfulness and psychological well-being. Keune and Fortintos present mindfulness meditational practice in a non-clinical context as a basis for improved well-being and tested the influence of mindfulness training (MT) on smoking cessation and found evidence of efficacy.

### Research Materials

Deci and Ryan (1985) posit that people are autonomously motivated when they engage in an activity for reasons that are freely chosen and coherent with their value system. In contrast, controlled motivation pertains to behaviors that are induced by forces perceived to be external to the self. Therefore, self-determination is the degree to which a person is self-determined or self-motivated, rather than externally driven. The Ryan and Deci (2000b) Self-Determination Scale was applied in this study. It is a valid self-report instrument consisting of a 10-item scale, with two 5-item subscales, to assess participant’s self-awareness, perceived choice and self-determination functioning.

Mindfulness training (MT) has recently emerged as a therapeutic modality for behavior modification and has become a mainstream psychological construct. Mindfulness pertains to subjective conscious awareness and promotes moment-to-moment attentiveness (Schuman-Olivier et al., 2014). MT is considered as popular and has proven useful for helping people disengage from unhealthy habits and thought patterns through meditative practice and Headspace “take-10” is a positive psychology intervention introducing the concept of mindfulness meditation practice in a practical 10-day, 10-min podcast session per day program (Howells et al., 2014). The decision to use Headspace take-10 as the evidence-based self-help mobile intervention was two-fold. In an effort to replicate empirically based happiness research (Howells et al., 2014) and to provide further evidence to support human flourishing and wellbeing initiatives in mental health care. In addition, data were collected using the Mindful Attention Awareness Scale (MAAS) which is a valid self-report instrument consisting of 15 items, to assess dispositional mindfulness in daily life and unique quality of consciousness. With a Cronbach’s alpha = 0.96, MAAS has demonstrated reliability and validity in numerous studies (Brown and Ryan, 2003).

### Population

Replicating sample sizes in a previous randomized controlled trial (RCT) of smartphone-based mindfulness intervention by Howells et al. (2014) and a psycho-education study by Joice and Mercer (2010), 136 participants were recruited from the existing database of registered members with M4JAM. Membership of 80,000 plus was rated as broad-based and thus an appropriate and convenient platform to recruit population sample. Key demographic information relating to M4JAM users indicated that 68% were between the ages of 25 and 34 years, 53% were males, 47% were females, less than <4% were unemployed, and 40% preferred English.

In terms of current study, eligibility criteria required potential participants to be over the age of 18 with a valid South African identity number, registered on M4JAM compliant with South African legislative requirements and the Financial Intelligence Centre Act 38 of 2001 (FICA), and with daily access to a smartphone device.

The invitation to participate in research on M4JAM mobile platform returned a total of 817 non-duplicated volunteers within a 7-day period, from a database of 82,000 registered members. Potential participants were asked to complete the self-reported, Self-Determination Scale (SDS) to ascertain motivation orientation, perceived choice and self-awareness. Thousand SDS were posted on the mobile platform with a return of 817. A response rate of 81.7% was considered as good. Based on the cumulative scores of self-reported measures, the volunteers (*N* = 817) were apportioned into two groups: high self-determination (HSD) and low self-determination (LSD). Forty-seven percent of respondents were female (*n* = 382) and 53% were male (*n* = 435). Respondents ranged in age between 18 and 66 years (*M*_age_ *=* 29.63, SD = 8.3). Forty-six percent of respondents were between the ages of 25 and 34 years and only 1% was between 55 and 64 years. Of the total 817 respondents, 16.64% (*n* = 136) scored less than 30 to constitute the heteronomous group and 83.36% (*n* = 681) rated themselves above 30 to constitute the self-determined group. A sample of *n* = 68 participants were drawn from the HSD group and (*n* = 68) participants were drawn from the LSD group.

The demographic characteristics of recruited potential sample population were assessed in terms of age and gender. To hold research conditions constant, sample groups were matched in composition based on SDS score, age, and gender. Participants from LSD and HSD groups were randomly assigned to either (A) the experiment group with financial incentive condition or (B) the control group without incentive. The sample was heterogeneous regarding age and gender: the mean age in LSD group was 30.89 years with 45.59% males and 54.41% females and the mean age in HSD group was 30.76 years with 45.59% males and 54.41% females.

### Procedure

M4JAM uploaded all relevant research documents and instructions to the mobile platform; these included: the invitation to participate, essential information on how to participate, instructions on how to set up daily prompts, and the participant satisfaction survey. Real-time reporting and data quality validation were enabled.

All registered M4JAM users were invited to participate in the research project *via* the M4JAM platform. Potential participants were directed to the essential information sheet detailing research facts, eligibility criteria (i.e., over 18 years of age, with a South African identity number, registered on M4JAM, able to read and write in English, have daily access to a smartphone and not currently in therapy). Participants were required to give consent and had the right to withdraw at any time.

Once prospective participants volunteered and accepted the research “job” and were eligible in terms of inclusion criteria and not excluded, potential participants were then asked to complete the Self-Determination Scale (SDS) to ascertain the potential participant’s self-awareness, perceived choice and self-determination functioning.

### Participants

Based on Self-Determination Scale scores, all prospective participants (*N* = 817) were divided into either the low self-determination group or the high self-determination group. A sample of *N* = 136 participants, *n* = 68 for low self-determination and *n* = 68 for high self-determination, were drawn and randomly assigned to either group A, with the experimental condition treatment, that was with a R10.00 incentive per session for correctly answering two questions pertaining to task, or group B, a control group, without an experimental condition, that was without any financial incentive for correctly answering two questions pertaining to task. Participants with high SD score were randomly assigned to SD group A and group B, and participants with low SD score were randomly assigned to Heteronomy group A and B (Table 1).

**Table 1:** Sample groups.

Self-determination		Heteronomy	
Group A (n34)	Group B (n34)	Group A (n34)	Group B (n34)
Single incentive	Double incentive	Double disincentive	Single disincentive
Self-determination	Self-determination and financial reward	Low autonomy and no financial reward	Low autonomy and financial reward

Groups were asked to complete the MAAS which is a valid 15-item self-report instrument to assess dispositional mindfulness in daily life. Baseline tests were not incentivized in any group. Scores were recorded at baseline to measure any dispositional mindfulness effect post intervention and no financial incentive was offered to any group to complete pre- and post-MAAS tests.

Participants were instructed how to download Headspace take-10 app and instructed to follow 10-day program. M4JAM sent all participants, in all groups, daily prompts and two-question authentication. They were requested to complete follow-up post-intervention questionnaire. The MAAS established a measurement of change (if any) in dispositional mindfulness in daily life. Participants were asked to complete client satisfaction survey to measure participant’s satisfaction with daily intervention and to provide feedback of incentivized mobile psychology intervention to M4JAM.

To meet the British Psychological Society ethical requirements for conducting research with human participants, including issues of consent and confidentiality, the proposal for this research study was reviewed and received full approval by University of Liverpool’s Research Ethics Committee, for the Institute of Psychology, Health and Society. All participants were given study information before the study to inform their decisions to take part, and those that agreed to participate gave written consent

## Results

Analysis comparing the variance of engagement between the four groups tested the null hypothesis about the effects of extrinsic and intrinsic motivation factors on participants’ behavior. User app engagement (i.e., the number of intervention days completed out of 10) describes the dependent variable, and the different combination of two levels of self-determination with or without a financial incentive describes the independents. A second and separate analysis comparing the variance of dispositional mindfulness within-groups, measured the before- and after-intervention scores of participant’s mindfulness attention awareness.

### Analysis of User App Engagement

As indicated in Table 2, (*n* = 136) participants divided into four equal groups participated in experiment. Total user app engagement averaged (*M*_days_ = 2.60, SD = 4.10). An alpha level of 0.5 was used for all statistical tests. Levene’s test of equality of error variances indicated that homogeneity of variance had been satisfied and that the error variance of the dependent variable, user app engagement, was equal across groups (*p* < 0.05).

**Table 2:** User app engagement.

Groups	*M*	SD
Low self-determination with no incentive (LSDA)	0.53	1.88
Low self-determination with incentive (LSDB)	4.29	4.79
High self-determination with no incentive (HSDA)	0.59	1.33
High self-determination with incentive (HSDB)	5.00	4.83
Total	2.60	4.11

**Figure 1:**
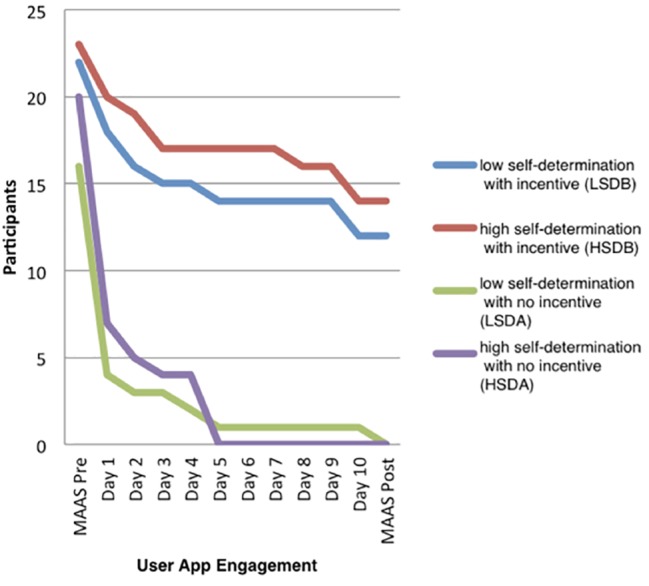
Participation over time.

General linear model, univariate analysis of variance presented the main effect and joint effects of the independent variables, self-determination, and financial incentive on the dependent variable, user app engagement *F*(3, 132) = 14.91, *p* < 0.01, $\eta_{p}^{2}$ = 0.253. Tukey’s *post hoc* testing demonstrated that the financial incentives drove user app engagement (*p* < 0.01) over and above self-determination. This provides evidence that there was a difference between incentivized and non-incentivized groups and that, in this case, the variable, financial incentive (*p* < 0.01, $\eta_{p}^{2}$= 0.25), influenced the dependent variable, user app engagement. Figure 1 illustrates the drop off in participation within the four groups and explains why only two groups were analyzed for the change in the dependent variable, dispositional mindfulness.

The total number of participants (*N* = 26) who completed the Headspace take-10 intervention, with pre-test and post-test MAAS, distributed across the groups as follows: LSDA (*n* = 0), LSDB (*n* = 12), HSDA (*n* = 0), and HSDB (*n* = 14).

**Figure 2:**
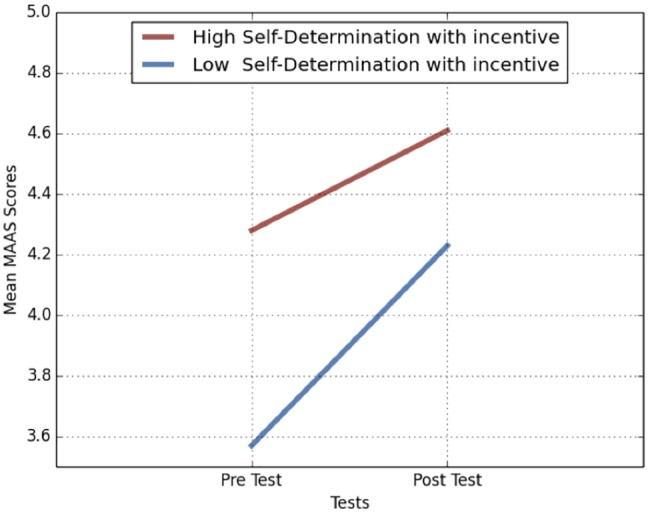
Mean MAAS scores of group participants.

An analysis of variance was conducted to measure the change in the dependent variable, dispositional mindfulness. Pre-test and post-test MAAS mean scores revealed that there was a difference between group means and within groups, as illustrated in Figure 2. However, the results were not significant in the high self-determination with incentive (HSDB) group. Pre-test revealed *F*(1, 24) = 7.120 and *p* < 0.05 and post-test revealed *F*(1, 24) = 2.158 and *p* > 0.05. At completion of the study, a *post hoc* power analysis was conducted and *r* = 0.1 and *observed p* = 0.191 were obtained, supporting low power “after the fact” (Pezzullo, 2015) which means that the sample size needed to be larger for meaningful analysis.

## Discussion

The purpose of this study was to evaluate any significant difference in mobile app user engagement in groups of individuals with self-reported measures of high self-determination, or self-reported measures of low self-determination when financial incentives were offered. This study was an opportunity to explore the viability of incentivized mobile psychology on mental health issues in South Africa and builds on papers reporting on the function of other mobile electronic device projects reported by Smith (2012). Overall, the findings supported an interaction between an extrinsic incentive contingent and positive psychology app engagement.

### The Dependent Variable “User Engagement” and Ecological Validity

Recent work describes user engagement as a multifaceted and positive interaction with an online application and information technology (Lalmas et al., 2015). The user invests time and attention in a technology resource without mediation. User engagement differs from user experience, in that user engagement represents not only the quality of the interaction but also volitional choice. The current study measured user engagement in terms of the number of correct answers and did not account for user repeating a session at a future point in time or digitally sharing experiences with others or returning to a session. Thus, user app engagement related to use in a real-life setting, and for the purpose of quantified data collection, was operationalized in measured units. Engagement and completion contingent rewards were given for completing a task in the experimental group. Similar to work by Vansteenkiste et al. (2012), the real-life experiment of testing engagement and volitional choice in real-world domain increased the study’s ecological validity.

### The Framework of Study – Self-Determination Theory

At the risk of oversimplifying the theory, self-determination, as coined by Deci and Ryan (1985) is characteristic of internally motivated behavior and, at a basic level, is defined as an individual’s capacity to choose and have choices to engage in activities without external influence, control, or reinforcement. The optimal type of motivation is that which is derived from the self, with autonomous regulation and functioning correlates with more positive consequences than controlled functioning and an external locus of control. It is maintained within SDT that autonomy, competence, and relatedness combined are fundamental psychological needs that energize behavior (Sheldon and Niemiec, 2006; Vansteenkiste et al., 2012).

The current study investigated the distinction between autonomous and controlled types of motivation, causality orientation and regulatory style. In principle, SDS is based on a continuum (Ryan and Deci, 2000a). On one side of the continuum is amotivation, which is absent of self-determined motivation, i.e., is non-self-determined, and is neither extrinsic nor intrinsic, to a high level of self-determination, which is both an intrinsic and integrated quality motivation on the other side of the spectrum, as explicated in article by Vallerand et al. (2008). Theoretically, a continuum cannot be separated into two discrete sets; however, in the pursuit of knowledge in this study, participants’ self-determination was scored, and two dialectical sets were discerned of high and low levels of self-determination: LSD (*M*_score_ = 27, SD = 3.022) and HSD (*M*_score_ = 41.72, SD = 4.74). At the risk of compromising internal validity, there was the likelihood that groups were more similar than discrete, which might have accounted for the non-significant effect in the interpretation of the self-determination results. However, even with a threat of measurement error and bias, the scale was useful to gauge and distinguish the participant’s motivation orientation. Lalmas et al. (2015) warned that all studies have different constraints and suggested mixed methods to improve validity and rigor. Vallerand et al. (2008) concur that little data exist regarding motivational change and placement of motivational type on the self-determination continuum.

User app engagement results suggest that financial incentive manipulation significantly increased user app engagement, over and above the impact of self-regulation and that there was a change in dispositional mindfulness in the low self-determination group, but not with the high self-determination incentive group. Participant’s self-reported measures of mindfulness were based on subjective interpretation and individual perception of mindfulness. Although MAAS is a valid instrument (*α* ≥ 0.80), there was a consideration, in this case, that participants may have differed in their construct of mindfulness and that responses were potentially biased. Research replicated the previous study by Howells et al. (2014) and applied the same evidence-based mindfulness course, Headspace take-10. By design, the mindfulness intervention was brief and introductory. It can, therefore, be assumed that any predicted change in dispositional mindfulness was also potentially limited. As a preliminary proof of concept, the current study demonstrated that incentivized mobile psychology, as a mental health initiative was possible. Our results suggest that people with high SD, with a financial incentive, were more likely to engage in a mobile psychology app than people with low SD with no financial incentive. However, the results indicated that in all groups, the financial incentive contributed significantly to user app engagement.

### Attrition

Attrition was common in all four groups and followed similar disengagement trends in each group. One possible interpretation of this factor can be drawn from the online dominance and attrition study by Sonntag and Zizzo (2015) who suggested that the size of the financial incentive should be correct in order to motivate engagement. Here, R10.00 may not have been a realistic incentive for sustaining task engagement. Similarly, the outcome of Lynagh et al. (2013) review of financial incentive for health behavior change found that a pay-for-performance approach was an effective and conventional treatment for disadvantaged groups, thus introducing the influence of socio-economic complexities, and validated that there is not a “one size fits all” solution. Future research is therefore required to evaluate different incentive features and structures and the influence on engagement in relation to socio-economic status and also consider the impact of realistic differential rewards and tailor alternative strategies. Here the daily intervention evaluation could have been used to understand the failure to engage and predict attrition rate. Attrition rate correlated with common trends in other incentive-based programs. Patel et al. (2011) similarly reported a drop off over time, i.e., that engagement declined after initial short-term participation.

## Conclusion

According to Lu (2015), internet-delivered psychological interventions aimed at mental health and behavioral improvements have become an acceptable alternative to traditional methods of therapy because young adults are more likely to search for online interventions than seek professional help. Given the exponential growth of cellular usage worldwide, and in particular, the increase in mobile users in sub-Saharan Africa, positive psychology interventions on a mobile platform have the potential to become an essential part of primary care in mental health services. With the convenience of use, the economic viability, and accessibility, Internet-delivered therapy has an advantage over other forms of mental health care. Convenience and accessibility are specifically relevant for vulnerable populations in developing countries, disadvantaged by geographical isolation, and a fundamental lack of resources. The current study could assist future policymakers in South Africa to consider the value of incentivized smartphone-based interventions and relevant mobile psycho-educational initiatives that could promote mental health literacy and fundamentally reduce mental health disparities and waiting list issues.

## Data Availability

All datasets generated for this study are included in the manuscript and/or the supplementary files.

## Ethics Statement

To meet the British Psychological Society ethical requirements for conducting research with human participants, including issues of consent and confidentiality, the proposal for this research study qualified for full approval by the University of Liverpool’s Research Ethics Committee.

## Author Contributions

CV, an MSc Student, collected the data and wrote the dissertation. AF supervised and crafted the paper.

### Conflict of Interest Statement

The authors declare that the research was conducted in the absence of any commercial or financial relationships that could be construed as a potential conflict of interest.
